# Purchasing power of civil servant health workers in Mozambique

**Published:** 2012-01-24

**Authors:** Fátima Ferrinho, Marta Amaral, Giuliano Russo, Paulo Ferrinho

**Affiliations:** 1 Associação para o Desenvolvimento e Cooperação Garcia de Orta, Lisbon, Portugal; 2 International Health & Biostatistics Department and Centro de Malária e outras Doenças Tropicais, Instituto de Higiene e Medicina Tropical, Universidade Nova de Lisboa, Lisbon, Portugal

**Keywords:** Purchasing power, civil servants, low-income countries, human resources for health, Mozambique, health sector, salary

## Abstract

**Background:**

Health workers’ purchasing power is an important consideration in the development of strategies for health workforce development. This work explores the purchasing power variation of Mozambican public sector health workers, between 1999 and 2007. In general, the calculated purchasing power increased for most careers under study, and the highest percentage increase was observed for the lowest remuneration careers, contributing in this way for a relative reduction in the difference between the higher and the lower salaries.

**Methods:**

This was done through a simple and easy-to-apply methodology to estimate salaries’ capitalization rate, by means of the accumulated inflation rate, after taking wage revisions into account. All the career categories in the Ministry of Health and affiliated public sector institutions were considered.

**Results:**

Health workers’ purchasing power is an important consideration in the development of strategies for health workforce development. This work explores the purchasing power variation of Mozambican public sector health workers, between 1999 and 2007. In general, the calculated purchasing power increased for most careers under study, and the highest percentage increase was observed for the lowest remuneration careers, contributing in this way for a relative reduction in the difference between the higher and the lower salaries.

**Conclusion:**

These results seem to contradict a commonly held assumption that health sector pay has deteriorated over the years, and with substantial damage for the poorest. Further studies appear to be needed to design a more accurate methodology to better understand the evolution and impact of public sector health workers’ remunerations across the years.

## Background

In Mozambique, as in many other developing countries, anecdotic evidence suggests that the purchasing power of civil servants in general and of civil servant health workers in particular, erodes over time. According to Reis et al. in Mozambique the salaries of civil servant health workers are defined by law, and these salaries have grown at rates lower than inflation [1]. Like for the majority of African countries [2], between 1991 and 1996, monthly salaries of nurses went from 110$ to less than 40$ and the salaries of doctors decreased from 350$ to 110$ [3]. Schwalbach and colleagues refer that health workers themselves identify salary increases as one of their most adamant demands [4].

The erosion of salaries’ purchasing power eventually becomes a source of dissatisfaction and of loss of motivation, leading to reduced productivity, loss of service quality, deterioration of the client-health workers interaction and illegal charges and petty corruption [5–8]. In the context of the development of a new human resources national development strategy for the Ministry of Health in Mozambique (for the period 2008-2015) this issue could not be ignored [9]. Therefore the variation in purchasing power between 1999 and 2007 was studied in order to refine recommendations regarding salary strategies.

Mozambique is to these days one of the world’ least developed country [10], and one with the worst health indicators [11]. Emerged in 1992 from a 16-year long civil war, its health care system has since gone through several phases of rehabilitation. While in the 1980s was explicitly focused on primary care-centred policies to expand access to services for its impoverished inhabitants [12], a transition to a more sophisticated health care model has recently been taking place, as the country's middle class and private sector steadily grows in urban areas, and the policy focus is gradually being moved towards the quality of services [13].

At the time of the study, the National Health Care System (NHS) relied on approximately 1,200 health units and 25,683 health personnel, among which only 2.5% were national doctors, and over 85% were little-trained “elementary” and “basic” cadres [14]. Ferrinho and Omar maintain that health workers’ large unskilled base, as well as their geographical distribution skewed towards the country's rural areas, are the health workforce main weak points [15].

Due to language and traditional barriers, Mozambican health professionals do not seem to be overly affected by international brain drain [16]. To this respect, Bhargava and Docquier estimated the medical brain drain in the country at around 6% of the workforce, down from its 7.5% peak in the nineties [17].

On the other hand, public sector health workers true brain drain appears to be directed towards the consolidating urban areas’ private sector. Although official statistics on the private sector are lacking, several studies have reported an ever-increasing proportion of public sector staff engaging in dual employment in private clinics, in their own practices or informally, a controversial if largely unregulated phenomenon in the country [18].

## Methods

The present work aims, firstly, at producing original data on health professionals’ salaries in Mozambique. Secondly, the paper's objective is to contribute to the development of a standardised methodology, easy-to-apply methodology to assess salary's purchasing power across the years in information-poor settings. Our study focuses on basic salaries in the health sector between 1999 and 2007, as these, together with salaries in the Education sector, have been the subject of the Mozambican Government's policies in the last decade aimed at recruiting and retaining key workers in the health sector. Allowances and salary supplements were not considered in the study as these, according to Tyrrell 2008, represent only a fraction (about 5.5%) of the official pay in the health secrtor in Mozambique [19]. Such salary supplements are also particularly unevenly distributed among professional categories in the Mozambican public sector [20], and it is here believed that establishing in what measure each civil servant benefitted from such extra pay would constitute the subject of a different study.

With the objective of calculating health sector salaries purchasing power, 1999 to 2007 salaries in the national currency (Mozambican Meticais – MZM) were obtained for civil servant health workers of the different careers (including managerial categories) in public sector health facilities and in the Ministry of Health [21].

Public sector careers in Mozambique are established by legislation as general, specific and special regime careers [22]. Special Differentiated Health Careers (*Carreiras Diferenciadas de Regime Especial da Saúde*), include the public health medical career, the hospital medical careers and the generalist medical career.

Special Undifferentiated Health Careers (*Carreiras Não Diferenciadas de Regime Especial da Saúde*) include the careers of university trained non-medical specialists, N1 and N2 university trained health technicians, specialized mid-level health technicians, health technicians, assistant health technicians and auxiliary health technicians (*Especialistas; Técnicos Superiores de Saúde N1; Técnicos Superiores de Saúde N2; Técnicos Especializados de Saúde; Técnicos de Saúde; Assistentes Técnicos de Saúde e Auxiliares Técnicos de Saúde*). General regime careers integrate occupations common to all sectors. The General Career Regime (Carreiras do Regime Geral) includes N1 and N2 university trained technicians, professional technicians, technicians, assistant technicians, auxiliary administrative personnel, manual workers, ancillary personnel and auxiliaries (*Técnicos Superiores N1; Técnicos Superiores N2; Técnicos Profissionais; Técnicos; Assistentes Técnicos; Auxiliares Administrativos; Operários; Agentes de Serviços e Auxiliares*). Health workers may still move to a different salary scale by assuming managerial positions.

The Mozambican currency (Metical: MZM) changed to a new currency, the Metical of the “New Family” (ISO 4217: MZN) on the 1st of July of 2006 (Law 7/2005, of 20th of December) where 1,000 MZM = 1 of the MZN. In this article wages are processed as MZM rather than MZN.

### Estimation of the capitalization rate

Health sector salaries have been revised upwards several times by the Government between 1999-2007, with the objective to keep health workers purchasing power on the face of an unpredictable inflation rate. Using a standard capitalization rate (CR) methodology from mathematical economics and financing [23, 24], we used the salary's capitalization rate as a means to compare 1999 health workers salary's purchasing power with its real value in 2007.

The CR was calculated on the accumulated inflation rate, and represented the “theoretical percentage increase” by which 1999 salaries should have grown until 2007 in order to maintain their original purchasing power. For those professional categories for which the 2007 “real” salaries were found to be higher than the “theoretical increase of 1999 wages” calculated through the capitalization rate, it was concluded that their salaries had actually lost purchasing power [25].

Following the standard approach to calculate the accumulated inflation rate (AIR) [26], we first estimated the yearly inflation rate index (IRI) for each n year, where n= 1999 … 2007:

IRI for year n = IR_(for year n)_/100;

Thereafter we estimated the accumulated inflation rate (variation):

Var accumulated inflation rate (AIR_1999–2007_) = (1+IRI_1999_)) X (1+IRI_2000_)) X (1+IRI_2001_)) X … (1+IRI_2007_));

The Var AIR_1999-2007_ for Mozambique was estimated to be 2.45.

On the basis of the AIR we estimated the CR, which is supposed to measure the proportional salary variation between 1999 and 2007. Considering “I_1999-2007_” as the absolute variation between salaries in 1999 (S_1999_) and salaries in 2007 (S_2007_), we applied the following permutations:

(1) I_1999-2007_/S_1999_ = I_2007-1999_/S_1999_

(2) S_2000_ = S_1999_(1+IRI_1999_)

S_2001_ = S_2000_(1+IRI_2000_)

S_2002_ = S_2001_(1+IRI_2001_)

.......

S_2007_ = S_2006_(1+IRI_2006_)

Then we substituted out (1) for (2) to obtain:

(3) I_1999-2007_/S_1999_ = S_1999_[(1+IRI_1999_)(1+IRI_2000_)(1+IRI_2001_)….(1+IRI_2007_)-1]/S_1999_

Where S_1999_ cancels out to arrive at the final formula for CR:

(4) CR_1999-2007_ = ( ([Var] AIR_1999-2007_) –1).

In percentage terms, CR_1999-2007_ for Mozambique for our study was calculated to be 145.38%.

The official national inflation rate as calculated by the National Institute of Statistics between 1999 and 2007 was used in our CR calculations.

It is recognised that the adopted CR methodology is based on a crude estimate of national inflation rates, which is not sensitive to regional (north-south as well as urban-rural) variations, or to price variation across different baskets of goods across the years. Data processing also resulted in extensive tables that were necessary to summarize by:

Limiting the presentation of results to the medians of: the monthly wages in each category; the accumulated percentage of the wage revisions observed yearly between 1999 and 2007; nominal wage adjustments for the period under study; real wage adjustment for the period; Presenting only the results for years of 1999 and 2007, however the salaries for every year of the period have been considered.

## Results

Table 1 specifies the 1999 and 2007 median monthly salaries (in the national currency, meticais – MZM) of health workers from the Special Differentiated Health Careers. For these careers, in 1999, the salaries varied between 4,739,328 MZM and 5,246,208 MZM and, in 2007, between 12,207,000 MZM and 13,513,000 MZM. This table also illustrates the median monthly salaries of health workers from the Special Undifferentiated Health Careers. Their lowest salary in 1999 was 822,960 MZM and the highest 5,246,208 MZM and, in 2007, 2,426,000 MZM and 13,513,000 MZM respectively. For this group of undifferentiated careers the highest salaries are similar to the highest salaries of the Special Differentiated Health Careers. Table 1's most interesting finding is that between 1999-2007, while salary purchasing power increased of around eight times for the majority of the careers considered, it increased 30-fold for the lowest-paid undifferentiated professionals.

**Table 1:** 1999–2007 evolution of the purchasing power of health workers from the Special Differentiated Health Careers and the Special Undifferentiated Health Careers

Health Careers	Monthly Salaries (MZM)*		Wage Revisions	Salaries: Nominal Wage Adjustment	Salaries: Real Wage Adjustment	Increase / Decrease in Purchasing Power of Health Workers	
	
	1999	2007	(1997–2007) (%)	(1999–2007)	(1999–2007)	(1999–2007) (%)	(1999–2007)
**Special Differentiated Health Careers**							
Public Health Medical	5,246,208	13,513,000	157.58	7,626,707	8,266,792	8.39	640,085
Hospital Medical	5,246,208	13,513,000	157.58	7,626,707	8,266,792	8.39	640,085
Generalist Medical	4,739,328	12,207,000	157.58	6,889,827	7.467.672	8.39	577,845
**Special Undifferentiated Health Careers**							
University Trained Specialists	5,246,208	13,513,000	157.58	7,626,707	8,266,792	8.39	640,085
University Trained Health Technicians N1	4,055,040	10,445,000	157.58	5,895,039	6,389,960	8.40	494,921
University Trained Health Technicians N2	3,345,408	8,617,000	157.58	4,863,407	3,345,408	8.39	408,185
Specialized Mid-level Health Technicians	2,018,016	5,199,000	157.65	2,933,703	3,180,984	8.43	247,281

MZM: Mozambican Metical (Mozambican currency)

Table 2 addresses the median monthly salaries of the civil servant health workers in the General Career Regime. The salaries for these careers varied between 450,000 MZM and 4,464,000 MZM in 1999 and 1,645,000 MZM and 11,560,000 MZM in 2007. It is noticeable that also for general career regime cadres, the salary purchasing power increase was much greater for unskilled low-pay assistants (between 44 and 83 times the 1999 level), rather than for university or professionally trained technicians (around 8 times). Professional technicians were the only category that did not see its salary purchasing power increased between 1999 and 2007, when its calculated power actually decreased by 11.78%.

**Table 2:** 1999–2007 evolution of the purchasing power of civil servant health workers in the General Career Regime

General Career Regime	Monthly Salaries (MZM)*		Wage Revisions	Salaries: Nominal Wage Adjustment	Salaries: Real Wage Adjustment	Increase / Decrease in Purchasing Power of Health Workers	
	
	1999	2007	(1997–2007) (%)	(1999–2007)	(1999–2007)	(1999–2007) (%)	(1999–2007)
University Trained Technicians N1	3,456,000	8,961,000	159.04	5,024,181	5,505,000	9.40	480,819
University Trained Technicians N2	2,682,000	6,953,000	159.20	3,898,974	4,271,000	9.51	372,026
Professional Technicians	1,908,000	4,354,000	128.24	2,773,767	2,446,000	−11.78	−279,373
Technicians	1,480,500	3,825,000	159.17	2,152,283	2,344,500	9.49	198,801
Assistant Technicians	1,053,000	2,113,000	159.57	1,530,805	1,673,000	9.76	143,315
Auxiliary Administrative Personnel	783,000	2,408,000	209.30	1,138,291	1,625,000	43.97	489,044
Manual Workers	720,000	2,218,000	209.30	1,046,704	1,046,704	43.97	451,296
Ancillary Personnel	661,500	2,863,000	235.83	961,660	1,559,500	62.22	597,840
Auxiliary	607,500	2,221,000	265.58	883,157	1,613,500	82.68	730,343

* MZM: Mozambican Metical (Mozambican currency)

Table 3 specifies the 1999 and 2007 median monthly salaries of health workers in managerial positions (*funç[otilde]es de Direcção e Chefia*). For health managers, in 1999, the salaries varied between 1,184,000 MZM and 7,400,000 MZM; and, in 2007, between 4,385,000 MZM and 19,066,000 MZM. In line with what shown in the previous tabulations, the career with the lowest salary (7^th^ group) experienced the highest increase in its purchasing power, while the career with the highest salary experimented a moderated increase, although superior to the median increase of the purchasing power of all Health System Managers (748,956 MZM).

**Table 3:** 1999–2007 evolution of the purchasing power of health system managers

Health Careers		Monthly Salaries (MZM)*		Wage Revisions	Salaries: Nominal Wage Adjustment	Salaries: Real Wage Adjustment	Increase / Decrease in Purchasing Power of Health Workers	
	
		1999	2007	(1997–2007) (%)	(1999–2007)	(1999–2007)	(1999–2007) (%)	(1999–2007)
2nd group	100	7,400,000	19,066,000	157.65	10,757,795	11,666,000	8.44	908,205
Subgroup of the 2nd group	85	6,290,000	16,206,000	157.65	9,144,126	9,916,000	8.44	771,874
3rd group	65	4,810,000	12,393,000	157.65	6,992,567	7,583,000	8.44	590,433
Subgroup of the 3rd group	55	4,070,000	10,486,000	157.64	5,916,787	6,416,000	8.44	499,213
4th group	45	3,330,000	8,580,000	157.66	4,841,008	5,250,000	8.45	408,992
Subgroup of the 4th group	40	2,812,000	7,626,000	171.19	4,087,962	4,814,000	17.76	726,038
5th group	36	2,442,000	6,864,000	181.08	3,550,072	4,422,000	24.56	871,928
6th group	30	1,776,000	5,720,000	222.07	2,581,871	3,944,000	52.76	1,362,129
7th group	23	1,184,000	4,385,000	270.35	1,721,247	3,201,000	85.97	1,479,753

* MZM: Mozambican Metical (Mozambican currency)

For the period under study the capitalization rate was calculated to be 145.38% for the careers considered in tables Table 1 to 3. As it was positive it means that the purchasing power would have decreased if it had not been corrected through wage revisions, which varied, for the different careers, between 127.71% and 270.35%.

The variable “increase/decrease of purchasing power of health workers” reflects the difference (in MZM) between the “real” (decided by the Government) and the “nominal” (determined by inflation) wage adjustments. With the exception of professional technicians in the General Careers Regime (Table 2), all the other health workers saw their purchasing power increased in 2007 compared to 1999.

Table 4 shows the calculated real wage gaps between lowest and highest paid workers for the four broad categories considered, in absolute as well as in proportional terms. As the highest purchasing power increases were observed for the lowest paid careers, it would be possible that this could have contributed to a reduction of the gap between the highest and the lowest salaries among health workers in Mozambique. The gap appeared considerable when considering all the salaries in the health sector, with the lowest pay representing 8.63% of the highest pay in 2007 (6.08% in 1999).

**Table 4:** Gap between lowest and highest salaries of health workers, globally and per career group

Career and management positions	Real wages (MZM)*				Absolute wage differences (MZM)		Lowest salaries as proportion of the highest salary (%)	
	
	1999		2007		1999	2007	1999	2007
	
	highest	lowest	highest	lowest	highest	lowest	highest	lowest
Special differentiated health regime careers	6,488,064	3,345,408	16,712,000	8,617,000	3,142,656	8,095,000	51.56	51.56
Special undifferentiated health regime careers	6,488,064	648,000	16,712,000	1,910,000	5,840,964	14,802,000	9.99	11.43
General regime careers	4,464,000	450,000	11,560,000	1,645,000	4,014,000	9,915,000	10.08	14.23
Management positions	7,400,000	1,184,000	19,066,000	4,385,000	6,216,000	14,681,000	16.00	23.00
Global	7,400,000	450,000	19,066,000	1,645,000	6,950,000	17,421,000	6.08	8.63

* MZM: Mozambican Metical (Mozambican currency)

As reflected in Table 4, although the absolute wage differences increased for all career groups, in 2007 the lowest salaries represented a higher proportion of the highest salaries, indicating a relative gap reduction for all career groups, except for the Differentiated Special Health Regime careers, where the proportion remained stable at 51.56%.

## Discussion

This study contributes to shed light on an issue considered as critical for human resources retention policies in the health sector, that of the erosion of salary purchasing power over the years. To calculate salary purchasing power the study adopted a simple standard methodology based on the accumulated inflation rate calculated by the National Institute of Statistics for the whole country for the period of interest. There is a recognition that such an approach may be at times simplistic, as inflation rates may not be able to pick up the whole impact of price variation across different regions or different basket of goods. The study's main limitation appears to be that it ignores the many salary complements that in practice seem to favour most the highest paid and those in management positions [27]. If these were considered the equity gains would, most probably, be attenuated. It is nonetheless believed that the methodology developed and the respective findings can be rather useful for policy-makers in Africa, as they manage to give an indication of salaries’ purchasing power variations across the years.

Our calculations suggest that health worker's salary have more than kept its purchasing power over the 1997-2006 period, which is to some extent at odds with what happened to other parts of Africa over the same period or to Mozambique in the previous decades. On the one hand, such results could be explained by the Government's policies in key social sectors following its Poverty Reduction Plan's commitment. On the other hand, it needs to be considered that the increase of health wages in Mozambique started from an extremely low basis, which allowed a comparatively affordable defense of real wages for selected sectors. Secondly, managing to keep inflation under control was probably the Government's monetary policy's main accomplishment in the period under consideration – yearly inflation was mostly kept single-digit between 1997 and 2006. Because of lack of data it is not possible to compare our purchasing power results with those of other countries. But, for 1999-2007, in the different job markets of Portuguese Speaking African Countries, the capitalization rate for public servants health workers’ wages was estimated as varying between 18.07% for Cape Verde, 20.64% for Guinea-Bissau, 218.53 % for S. Tomé and Prince and 34675.88 % for Angola, giving an indication of the financial effort required from the different Governments to update the purchasing power of their health sector employees [28].

The study results also appear to reflect the positive impact of the evolution of the macroeconomic frame in the last few years. In fact, from 1997 to 2003 Mozambique registered a median economic growth of approximately 9%, a much higher value than the median of the African continent, and within a context of low inflation and increasing foreign direct investment [29].

The current System of Health Careers was approved in 1998 (Government law n° 64/98 of December the third) and the Ministry of Health was an active partner in the elaboration of the national Poverty Reduction Strategic Plan. A major purpose of this law was to retain in civil service the most differentiated professionals, by diminishing the gaps between the careers with the higher and the lower salaries. As observed in this study, this gap remains, and has increased in absolute terms, although the relative gap has decreased, i.e. the lowest salaries represent a higher percentage of the highest salaries in 2007 when compared with 1999. Although this might reflect a tendency for a more egalitarian society, it may also represent a tendency for salary compression, which may impact negatively on strategies to retain more differentiated personnel.

The salaries on non health-specific professionals appeared to be the only ones to effectively loose purchasing power in the period under consideration. If it may be to some extent understandable why a pro-health sector regulation may end up benefitting primarily physicians, nurses and health technicians, the unforeseen consequence was to make the health sector unattractive for managers and administrative personnel, all cadres essential for the organisation of the health system.

Our results suggest that a general improvement of the purchasing power of the public sector health workers was attained through successive wage revisions, but also through government effectiveness in keeping and sustaining the inflation rate at low levels. The extent to which this meets the wage expectations of different health worker groups is not clear and has not been studied. However, the fact that MoH training courses are generally oversubscribed, and that international brain-drain does not appear to be an imminent problem for the Mozambican health service, seems to suggest that health sector salaries are still regarded as appreciable, in the context of the scarce opportunities offered by the Mozambican labour market. It the other hand, it is also known that the private sector, as well organizations and projects offer better salaries and benefits, which appears to have led to a different kind of brain drain of the national health workers [30]. Further studies appear to be needed to design a more accurate, easy-to-apply methodology to help understand the evolution of health workers’ pay's purchasing power, and how this affects their decision to stay or leave the sector.

## Conclusion

The present study investigated the changes of health sector's civil servants salaries over ten years, to understand how their purchasing power was affected. To calculate salary purchasing power the study adopted a simple standard methodology based on the accumulated inflation rate calculated by the National Institute of Statistics for the whole country for the period of interest. Our calculations suggest that health worker's salary have more than kept its purchasing power over the 1997-2006 period, and that the salaries on non health-specific professionals were the only ones to effectively loose purchasing power in the period under consideration. Our interpretation was that a general improvement of the purchasing power of the public sector health workers was attained through successive wage revisions, but also through government effectiveness in keeping and sustaining the inflation rate at low levels.

The study showed the value of developing easy-to-apply methodologies to help understand the evolution of health workers’ pay's purchasing power, and how this affects their decision to stay or leave the sector.
